# Comprehensive Genomic Analysis of a *BRCA2* Deficient Human Pancreatic Cancer

**DOI:** 10.1371/journal.pone.0021639

**Published:** 2011-07-05

**Authors:** Louise J. Barber, Juan M. Rosa Rosa, Iwanka Kozarewa, Kerry Fenwick, Ioannis Assiotis, Costas Mitsopoulos, David Sims, Jarle Hakas, Marketa Zvelebil, Christopher J. Lord, Alan Ashworth

**Affiliations:** Breakthrough Breast Cancer Research Centre, The Institute of Cancer Research, London, United Kingdom; Sanford-Burnham Medical Research Institute, United States of America

## Abstract

Capan-1 is a well-characterised *BRCA2*-deficient human cell line isolated from a liver metastasis of a pancreatic adenocarcinoma. Here we report a genome-wide assessment of structural variations and high-depth exome characterization of single nucleotide variants and small insertion/deletions in Capan-1. To identify potential somatic and tumour-associated variations in the absence of a matched-normal cell line, we devised a novel method based on the analysis of HapMap samples. We demonstrate that Capan-1 has one of the most rearranged genomes sequenced to date. Furthermore, small insertions and deletions are detected more frequently in the context of short sequence repeats than in other genomes. We also identify a number of novel mutations that may represent genetic changes that have contributed to tumour progression. These data provide insight into the genomic effects of loss of BRCA2 function.

## Introduction

Individuals heterozygous for loss of function mutations in the tumour suppressor gene *BRCA2* are highly predisposed to a range of different cancers. Women that inherit a mutant *BRCA2* allele have a significantly highly elevated lifetime risk of developing breast and ovarian cancers [1]. Additionally, male breast and prostate cancers are strongly associated with *BRCA2* gene mutations [2]. In both sexes, *BRCA2* deficiency increases the risk of developing cancers of the pancreas, stomach, gallbladder, and bile duct, as well as melanoma [3]. The treatment of pancreatic cancer presents significant challenges as most patients present with locally advanced or metastatic disease and conventional anticancer therapies show limited effectiveness; only 5% of patients with pancreatic cancer survive beyond five years from the initial diagnosis [4], [5].

BRCA2 plays a key role in the maintenance of genomic integrity, particularly through regulation of DNA repair by homologous recombination repair (HR) [6] a process that is also controlled by another tumour suppressor protein, BRCA1 [7]. HR is a largely error-free process that restores the original sequence at the site of a DNA double-strand breaks (DSBs) [8]. DSBs arise relatively frequently and can be caused by normal cellular replication as well as exogenous stress such as exposure to ionising radiation [9]. In the absence of HR, for example due to loss of BRCA2 function, DSBs appear to be repaired by more error-prone processes that ultimately lead to the accumulation of gross chromosomal rearrangements [10]. It is thought that the utilisation of error-prone DNA repair processes in the absence of BRCA2 function most likely fosters tumourigenesis [10]. As part of its role in HR, BRCA2 controls the loading and removal of the DNA recombinase RAD51 at DSBs. The resulting RAD51-ssDNA filament mediates the search for a homologous DNA sequence to template the repair of the DSB [11].

Despite the great interest in BRCA2 function and its role in tumourigenesis and DNA repair, there are few tumour cell models of BRCA2 deficiency that can be used productively in the laboratory. Of these Capan-1 is the most well-characterised. Capan-1 was derived from a liver metastasis in a 40-year-old Caucasian male with a primary pancreatic adenocarcinoma [12], [13]. These cells lack a functional *BRCA2* allele and instead carry a *c.6174delT* allele. The single base deletion at *c.6174* causes a frameshift, (p.S1982fs*22) resulting in loss of the C-terminal 1416 amino acids of the protein [14], [15]. The resultant truncated protein lacks two BRC motifs involved in the interaction between BRCA2 with RAD51 and ssDNA [16], as well as C-terminal sequences thought to be required for nuclear localization of BRCA2 and RAD51/DNA disassembly [17], [18]. This truncated BRCA2 isoform has been shown to be both cytoplasmic and dysfunctional in HR [19], [20]. In keeping with the concept that BRCA2 dysfunction leads to genomic instability, SKY karyotype analysis has demonstrated that Capan-1 possesses a hypotriploid genome, with 36 defined structural rearrangements distributed across the entire genome [21]. The majority of these rearrangements are likely very complex and appear to involve more than three chromosome segments, although two reciprocal translocations (t(6;15) and t(7;10)) have been described (www.path.cam.ac.uk/~pawefish).

Given the considerable use of the Capan-1 cell line as a model not only of BRCA2 dysfunction but also of pancreatic cancer, we used next generation sequencing technology to study the genomic sequence of this cell line.

## Results

### DNA sequencing strategy

To identify candidate structural rearrangements we first generated a medium-depth whole genome sequence of Capan-1. We isolated DNA from a Capan-1 cells, and generated a 500 bp fragment DNA library using a PCR-free approach that improves library complexity [22]. This DNA library was sequenced using a paired-end strategy on an Illumina GAIIx Genome Analyser, yielding 365,811,868 raw 76 bp mate-paired reads (27.8 Gb). After alignment to a reference human genome (hg19/build37) and the subsequent filtration process to remove PCR duplicates and poor-quality reads, this data gave sufficient genome coverage (90.09%) for the study of structural rearrangements, at a median depth of 8.55-fold (Table 1; Table S1).

**Table 1:** Summary of sequence output.

	Whole genome	Exome
**Number of Flow-cell lanes**	14	3
**Insert size**	500 bp	200 bp
**Read lengths**	2×76 bp	2×76 bp
**Total reads**	365811868	130310847
**Mate-paired reads**	300129823 (82.04%)	116358031 (89.29%)
**Mate mapped to different Chr**	20579769 (5.63%)	2316964 (1.78%)
**Orphan reads**	37706652 (10.31%)	2968254 (2.28%)
**Reads on baited regions**	-	84694542 (65%)
**Median depth**	8.55	89
**Coverage**	2788939551 (90.09%)	37068745 (98.51%)
**Reference genome length**	3095677412	37629603

To investigate the coding sequence of Capan-1 in greater depth, we used a targeted enrichment strategy based on Agilent SureSelect in-solution capture. We utilized the SureSelect Human All Exon kit, designed to capture greater than 38 Mb of human genomic DNA corresponding to the NCBI Consensus CDS database. Two independent capture hybridizations were performed on approximately 200 bp fragments of Capan-1 genomic DNA. The resulting exome libraries were sequenced on an Illumina GAIIx using paired-end 2×76 bp sequencing runs. This yielded 130,310,847 raw reads (9.9 Gb). Similarly to the whole genome analysis, sequence reads from the capture process were aligned to a reference human genome (hg19/build37) using Burrows-Wheeler Aligner (BWA, [23]) and then filtered to remove PCR duplicates and poor-quality reads. The percentage of reads on target was 65%, reaching a final coverage of 98.51% for the baited regions with a median depth of 89-fold (Table 1; Table S2), far exceeding the 30-fold depth required to identify genetic variants in tumour samples [24].

For both the whole genome and exome sequencing strategies, more than 80% of reads were aligned in conjunction with their respective mate-pair (Table 1). In the case of the whole genome re-sequencing, 5.63% of reads mapped to a different chromosome compared to their mate-pair, and a further 10.31% of reads were orphaned, i.e. where the mate-pair failed to align due to excessive N or mismatches/indels (Table 1). This supports previous low-resolution spectral karyotype analyses (SKY) of Capan-1 that suggest a highly rearranged genome [21]. For the exome sequence, slightly fewer reads were aligned independently of their mate-pair than for whole-exome sequencing, perhaps suggesting that rearrangements within the coding region occurred less frequently in Capan-1 than in non-genic regions (Table 1).

**Figure 1:**
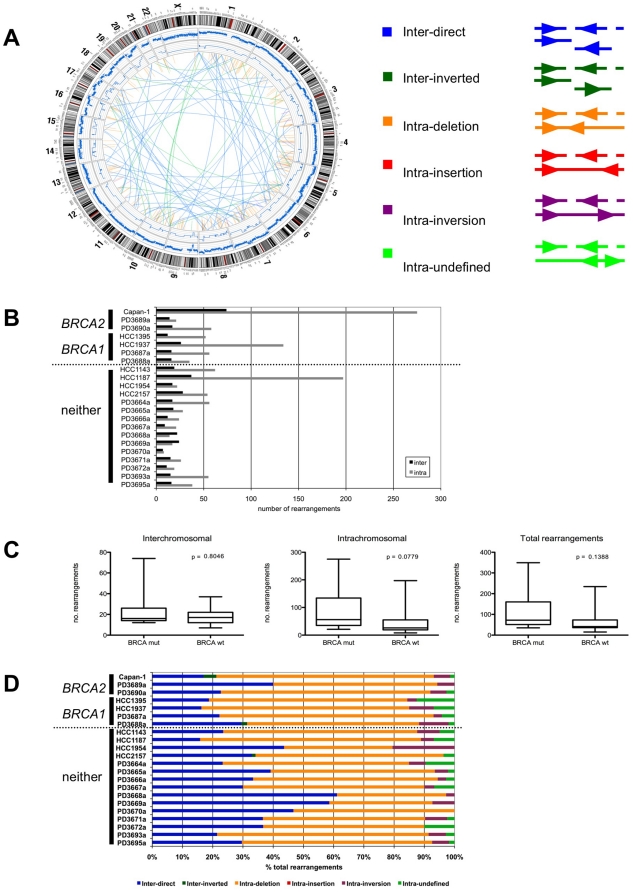
Structural rearrangements in Capan-1. **A.** Genome-wide Circos plot of the rearrangements identified in Capan-1 by BreakDancer analysis. An ideogram of a normal karyotype is shown in the outer ring, and copy number is represented by the blue line in the middle rings. The relative position and class of each rearrangement is depicted within the inner circle, as described in the legend. **B.** Comparison of the total number of inter- and intra-chromosomal rearrangements detected by BreakDancer analysis in a panel of *BRCA2*, *BRCA1*, and non-*BRCA* mutated cell lines and primary tumours. **C.** Samples were classified as *BRCA*-mutated (mut) or BRCA wild-type (wt), and the numbers of inter-, intra-chromosomal, and total rearrangements were compared. p values refer to Mann Whitney analysis with Gaussian approximation. **D.** Comparison of the percentage of each class of rearrangement in each sample. Samples are classified as *BRCA2*, *BRCA1*, and non-*BRCA* mutated, as in B.

Sequence depth across the genome was compared to data generated by array comparative genomic hybridization (aCGH) (Fig. S1, Table S1). Generally the median depth for each chromosome identified by sequencing matched the copy number profile generated by aCGH (Fig. S1). For example, chromosomes such as 4 and 6, that by aCGH appeared to be present in two copies, predominantly showed even depth across their whole length. In addition, the single copy X chromosome returned approximately half the number of reads of chromosomes 4 and 6. The previously reported [21], [25] homozygous deletion of 9p21 involving *CDKN2A*, and loss of the majority of chromosome Y, were also confirmed by sequencing (Figure 1A; Fig. S1, Table S1).

### Structural variation

To identify candidate structural rearrangements, we analysed the whole genome sequence of Capan-1 using BreakDancer [26]. We used a stringent filtering method that only identified rearrangements supported by at least ten reads (the median depth across the genome was 8.55x). This approach identified 354 large structural variations in Capan-1, which were sub-classified as intrachromosomal deletions, insertions, inversions, or inter-chromosomal direct or inverted translocations (Fig. 1A). No insertions were detected in this analysis, as they all fell within the boundaries of normal fragment size distribution (1–1000 bp).

Given that Capan-1 is a BRCA2 deficient model, we investigated the possibility that medium depth, whole genome sequencing could be used to distinguish BRCA1 and BRCA2 deficient tumours from non-familial forms. To date, two BRCA2 deficient tumours, two BRCA1 deficient tumours, and two BRCA1 deficient cell lines have also been subjected to medium-depth whole genome sequencing, as part of a wider study of primary breast tumours and cell lines [27]. We obtained the raw data from this study [27], and processed it through our own pipeline, which included analysis with BreakDancer. In this way, we were able to directly compare the frequency and type of structural rearrangements identified in Capan-1 with both BRCA deficient and proficient primary breast tumours and cell lines (Fig. 1B–D). Of all the genomes studied, Capan-1 exhibited the most structural rearrangements, both inter- and intrachromosomal (Fig. 1B). Although the sample number under study was relatively low (n = 7 *BRCA* mutant tumours vs. n = 15 non *BRCA* mutant tumours) comparison of the Capan-1 data with data from *BRCA2* mutant primary breast tumours and *BRCA1* mutant primary breast tumours and cell lines did suggest a trend for BRCA deficient samples to exhibit larger numbers of structural rearrangements, (median number for *BRCA* mutant tumours  = 72 rearrangements, median number for non-*BRCA* mutant tumours  =  41; Fig. 1C), consistent with the roles of BRCA1 and BRCA2 in maintaining genomic stability. Although this observation was not statistically significant (p  =  0.1368, Mann Whitney), this could be attributed to the low sample size. Moreover, as mutations in genes other than *BRCA1* and *BRCA2* affect HR, it is entirely possible that some of the samples classified as “non-*BRCA*” could also have a similar HR deficiency to *BRCA1/2* mutant tumours. The percentage of total rearrangements in each class was also compared across the *BRCA*-mutant and non-mutant groups (Fig. 1D). In Capan-1, as in the majority of the other genomes, intrachromosomal deletions were the most frequent class of rearrangement. However, there was no clear type or pattern of rearrangements that was specific to *BRCA*-mutant cells, based on this small set of samples.

A number of gross structural rearrangements have previously been identified in Capan-1 using spectral karyotype (SKY) analysis [21]. However, the resolution of SKY analysis is relatively low and in many cases the coordinate positions of many of the putative rearrangements identified with this form of analysis cannot be predicted with any great confidence. Given this, we only attempted a comparative analysis of NGS data with SKY data where the coordinate positions of SKY predicted rearrangements could be made within ∼10 Mb. This comparative analysis suggested that six of the previously identified rearrangements could also be identified using whole genome sequencing, but at higher resolution (Table 2). A further complex rearrangement between chromosomes 6, 8, and 17 had been previously suggested by the SKY analysis, although not in sufficient detail to elucidate the exact regions of each chromosome involved. Our analysis also suggested complex translocations between chromosomes 6 and 8, and between 8 and 17 (Table 2).

**Table 2:** Simple interchromosomal rearrangements.

SKY karyotype	Simple interchromosomal rearrangements	
*(Ghadimi et al. 1999)*	**Chromosome A**	**Chromosome B**
t(1;15)(q23;q15)	1∶180040326 (1q25.3)	15∶49200596 (15q21.2)
t(4∶15)(q26:?)	4∶123601213 (4q27)	15∶75679575 (15q24.3)
t(6;15)(q23;q21)	6∶119442950 (6q22.31)	15∶47336161 (15q21.1)
t(7;10)(q21;q24)	7∶61968749 (7q11.21)	10∶10951669 (10p14)
t(2;9)(?;q34)	2∶116377534 (2q14.1)	9∶131456807 (9q34.11)
t(5;10)(?;q24)	5∶134258528 (5q31.1)	10∶2348187 (10p15.3)
	**Complex interchromosomal rearrangements.**	
t(6;8;17)(p10;q10;?)	6∶58779888 (6p11)	8∶99469184 (8q22.2)
	8∶30144995 (p12)	17∶7168304 (p13.1)
	8∶118046478 (q24.11)	17∶57445353 (q23.2)
	8∶124144079 (q24.13)	17∶55207573 (q23.1)

Interestingly, eleven of the identified interchromosomal rearrangements identified in Capan-1 appeared to coincide with genic regions at both breakpoints, potentially suggesting gene fusion events (Table 3). None of these fusions have been previously reported in the Cancer Genome Census [28]. A further 42 rearrangements were found within a gene or upstream regulatory regions at one breakpoint, which could also have deleterious effects (Table S3). One of these rearrangements had a breakpoint in a gene, *ZNF521*, which was recently identified as the subject of chromosomal translocations in acute lymphoblastic leukaemia [29]. A further 118 genes appeared to be affected by intrachromosomal rearrangements (Table S4).

**Table 3:** Putative chromosomal rearrangements leading to gene fusions.

Chromosome A		Chromosome B	
Chr:Coordinate	Gene	Chr:Coordinate	Gene
1∶240318311	*PLD5*	5∶167115542	*ODZ2*
10∶127575097	*FANK1*	13∶20370053	*XPO4*
11∶10519362	*LOC100129827*	2∶149639082	*LYPD6B*
13∶110066008	*CARKD*	7∶145694385	*CNTNAP2*
13∶110066008	*CARKD*	2∶149639082	*LYPD6B*
15∶47235267	*GALK2*	6∶119442950	*FAM184A*
16∶76691051	*WWOX*	7∶127929929	*METTL2B*
17∶57374747	*MED13*	8∶118046478	*SLC30A8*
17∶7159674	*NEURL4*	8∶30144995	*DCTN6*
18∶2837027	*EMILIN2*	8∶100508052	*VPS13B*
20∶51993485	*BCAS1*	6∶88216683	*C6orf165*

### Exome single nucleotide variant (SNV) and Indel discovery

To identify SNVs and small insertion/deletion (indel) events in the coding sequence of Capan-1, we used SAMtools [30] for the pile-up analysis of the high-depth exome sequence and subsequent SNV calling. Small indels (1–6 bp) were identified using a custom pipeline (see Methods). Larger indel discovery was achieved using Pindel [31]. In the first instance, variants were filtered to exclude those detected at a depth of less than ten reads per copy. SNVs and indels referenced as general population polymorphisms in dbSNP were also disregarded, as we were largely interested in the identification of likely cancer-specific changes.

Like the majority of tumour-derived cell lines, Capan-1 does not have a matched normal cell line from the same patient that could be used to help identify cancer-specific changes and remove artefacts arising from both sample production and alignment. In light of this, we developed a process of filtration for exome resequencing, based on cross-comparison with data obtained from the analogous sample preparation and analysis of four normal genome HapMap samples (NA11881 (male); NA12761, NA12813, and NA12892 (female)) (Table S5). Using these HapMap samples, we were able to remove artefacts arising from both sample production (e.g. variation in hybridization efficiency) and alignment (e.g. homologous regions).

Analysis of the data obtained from exome resequencing of the HapMap samples enabled us to set thresholds for homozygosity (variant reads > 88% or < 10% total) and heterozygosity (variant reads 33–67% total) of variants. These thresholds were used to filter the identified SNVs and indels in the Capan-1 genome. Furthermore, as we were interested in identifying candidate tumour-specific mutations, we disregarded all mutations that were also detected in the HapMap samples, as an approximate normalisation for likely germline variation.

As a further level of stringency we also considered copy number changes across the Capan-1 genome, as defined by the aCGH analysis. This is particularly relevant for highly rearranged and aneuploid genomes, such as Capan-1. Based on our experience, the use of region-specific filters for sequencing depth facilitates more reliable variant identification. Clearly, heterozygous variants called in single copy regions are likely to be false (most likely due to misalignment), and elsewhere, the number of reads bearing the variant allele can fluctuate differently depending on the copy number status. Hence confidence thresholds were applied to both SNV and indel discovery, requiring a minimum of 10 reads per genomic copy. In addition, at least three variant reads were required to call a SNV and at least seven variant reads for indels, per genomic copy. The resulting catalogue of SNVs and indels identified in Capan-1 are detailed in Tables S6 and S8, respectively.

### Characterization of coding SNVs

Low throughput candidate gene sequencing studies in Capan-1 have previously identified three homozygous SNVs in *KRAS*, *SMAD4* and *MAP2K4*. Using exome sequencing, we were able to detect all three of these changes: the *KRAS* c.35G>T mutation that causes the clinically relevant p.G12V amino acid substitution [32], [33], the c.1028C>G SNV in *SMAD4* that results in a premature stop codon [34] and the *MAP2K4* c.661G>T mutation in Capan-1, which leads to a cancer-associated protein truncation [35]. The appearance of all three of these mutations in the final filtered list of exome variants, gave confidence to our analysis pipelines (Table S6).

Using high-depth exome sequencing, we identified a total of 608 SNVs in Capan-1 that affect 1270 different transcripts (Table 4). Most mutations (61%) were found in non-coding regions (non-coding genes or introns), or were synonymous variants. Twenty-four percent of the affected transcripts were modified by non-synonymous coding changes. In total, non-synonymous SNVs were detected in 206 different genes, 56 of which were homozygous (Table S6). Twelve genes gained premature stop codons, four of which were homozygous (*GRIA3*, *GRM1*, *MAP2K4*, *SMAD4*). Significantly, the number of probable somatic coding mutations detected for Capan-1 in this study compared very favourably with those shown in earlier studies examining the tumour cell lines COLO-829 (172 non-synonymous SNV) [36] and NCI-H209 (94 non-synonymous SNV) [24]. Both of these earlier studies identified somatic mutations by comparison with a matched normal blood DNA control from the same patient, suggesting that our use of HapMap exomes is an efficient alternative for the identification of somatic mutations in Capan-1, and other “orphan” cell lines.

**Table 4:** Predicted effects of SNVs on protein function.

	Exome
Novel SNVs passing filters (genes)	608
Total no. transcripts affected by SNVs	1270
Within non-coding gene	238
Intronic	280
Synonymous coding	257
Non-synonymous coding	303
Gained premature stop codon	12
Splice-site	87
5′-UTR	44
3′-UTR	49

**Figure 2:**
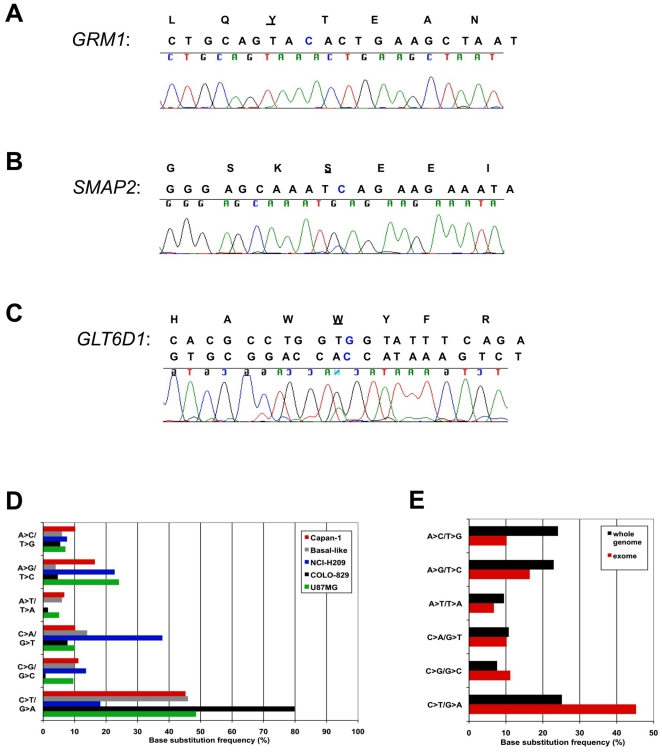
SNVs identified in Capan-1. **A.** Chromatogram depicting the stop-gain SNV in *GRM1* detected for Capan-1. The protein and genomic reference sequences are shown above the chromatogram. The SNV is highlighted in blue in the reference sequence, and the affected residue is underlined. **B.** As A, for *SMAP2*. **C.** As A, for *GLT6D1*. **D.** Comparison of the single base substitution frequencies in Capan-1, basal-like breast tumour, NCI-H209, COLO-829, and U87 MG genomes. **E.** Comparison of the whole genome and exome-specific base substitution frequencies observed in Capan-1.

Three of the novel potential truncating mutations were chosen for validation by Sanger sequencing: *GRM1* (*c.C1458A*, p.Y486*), *SMAP2* (*c.C764G*, p.S255*), and *GLT6D1* (*c.G593A*, p.W198*). All three were shown to be true SNVs, and the zygosity detected by the exome sequencing was also confirmed by Sanger sequencing: homozygous for *GRM1*, heterozygous for *SMAP2* and *GLT6D1* (Fig. 2A-C). *GRM1* encodes a metabotropic glutamate receptor, aberrant expression of which has been suggested to play a role in the development of melanoma [37]. SMAP2 is an ARF1-specific GTPase-activating protein involved in clathrin-dependent membrane trafficking [38]. The *GLT6D1* gene encodes an as-yet uncharacterised glycosyltransferase-6-domain-containing protein.

All of the novel SNVs identified here were cross-referenced with a number of online databases (SIFT [39], [40], Mutation Taster [41], [42], DAVID [43], [44], CGC [28], [45]) to predict their ability to modify protein function. One of the most interesting candidates was a homozygous c.*C162A* mutation in *FZD10*, a member of the frizzled gene family which encode Wnt ligand-binding receptors [46]. The homozygous *c.C162A* variant detected in this study leads to a non-synonymous amino acid substitution (p.N54K) at a highly conserved residue within the putative Wnt ligand-binding site (FZ domain) [47], [48]. FZD10 is a positive regulator of the Wnt-βCatenin-TCF signalling pathway, has been shown to be up-regulated in primary colorectal tumours [49], and Wnt-beta Catenin signalling is known to be aberrant in some pancreatic adenocarcinomas [50].

Three homozygous variants were present in the tumour suppressor *DCC* (*Deleted in Colorectal Carcinoma)*, which encodes a netrin receptor required for cell differentiation [51], and also the induction of apoptosis in the absence of ligand [52]. Mutations and loss of *DCC* have previously been implicated in pancreatic cancer, as well as a range of other tumour types [52], [53]. One of the three novel variants detected in Capan-1 was sited in the donor splice site of intron 20–21, four bases downstream of the critical GT motif. The other two variants gave rise to non-synonymous protein mutations (p.L1042 M and p.K1411T). Although neither of these latter non-synonymous mutations have been previously reported, both are sited close to residues of interest: p.F1039S, mutated in adenocarcinoma [54]; and p.Y1418, a critical residue phosphorylated by Fyn, and required for DCC function [55].

Cross-referencing of the SNVs detected in Capan-1 with the Cancer Gene Consensus (CGC) [28], [45] identified twelve genes that have previously been shown to be mutated at other residues in cancer cell lines or primary tumours. None of the variants identified in Capan-1 in this study have been previously described. However, the majority of these changes were non-coding or synonymous changes (Table S7). A heterozygous non-synonymous mutation was observed in the genome stability checkpoint factor *Ataxia Telangiectasia Mutated* (*ATM*). However, the variant residue (p.R1585S) lies outside of any known functional domains and it is therefore difficult to predict the functional consequences of this change. A second novel heterozygous non-synonymous mutation was identified in *TPR* (*Translocated Promoter Region*), which encodes a protein that interacts with the nuclear pore complex. This mutation (p.V779I) encompasses a conserved residue within a known motif common to chromosome segregation proteins, although the valine to isoleucine substitution identified here is likely to be neutral.

### Patterns of base substitution

We wanted to assess whether the pattern of single base substitutions in Capan-1 reflected that of other cancer genomes. The previously published human cell line genomes, COLO-829 [36], and NCI-H209 [24] each exhibit a characteristic pattern of base substitutions, representative of UV and tobacco carcinogen exposure, respectively. We obtained the data from these studies, in addition to that of the glioblastoma cell line U87 MG [56], and a basal-like breast tumour [57], for comparison with Capan-1. Comparative analysis of these sequences suggested that Capan-1 did not exhibit an obvious pattern of base substitutions or a unique signature representative of a particular mutagen. The majority of the coding substitutions (∼45%) in Capan-1 were C>T/G>A, although these were not as predominant as observed in COLO-829 cells where C>T transitions at dipyrimidine sites, indicative of UV exposure, predominate [36] (Fig. 2D). Based on the limited number of cancer genomes that have been sequenced to date, the pattern of base substitutions observed for Capan-1 most resembles that of U87 MG and the basal-like breast cancer, only differing significantly in the percentage of A>G/T>C mutations (Fig. 2D).

We also compared the pattern of base substitutions in Capan-1 in the entire genome versus the exome. We observed that the pattern was largely the same in coding and non-coding regions, except that C>T/G>A mutations were represented at a higher frequency in the exome, with concurrent lower numbers of A>C/T>G substitutions (Fig. 2E).

### Characterization of coding indels

As for the SNVs, novel indels identified in the Capan-1 cell line were filtered according to copy number. We noted that 93 genes were affected by small indels, ranging from one to six base-pairs (Table S8). More deletions were identified compared to insertions (Table 5), as would be expected from the use of gapped-alignment methods. 18 of the indels (nine insertions, nine deletions) were homozygous variants, whereas 62 were heterozygous (27 insertions, 35 deletions). Similarly to the SNVs, variation effects were classified according to transcript (163 across the 93 genes), and the majority of indels (72%) were detected in non-coding regions. Just 13% of indels caused frameshifts and 15% affected splice-sites. The hallmark *BRCA2 c.6174delT* mutation in Capan-1 [14], [15] was one of the 15 genes scored as being affected by frameshift coding deletions.

**Table 5:** Predicted effects of indels on protein function.

	Exome
**Novel indels passing filters (genes)**	**93**
Insertions	41
Deletions	52
**Total no. transcripts affected by indels**	**163**
Within non-coding gene	40
Intronic	68
Frameshift, coding gene	22
Splice-site	24
5′-UTR	2
3′-UTR	7

**Figure 3:**
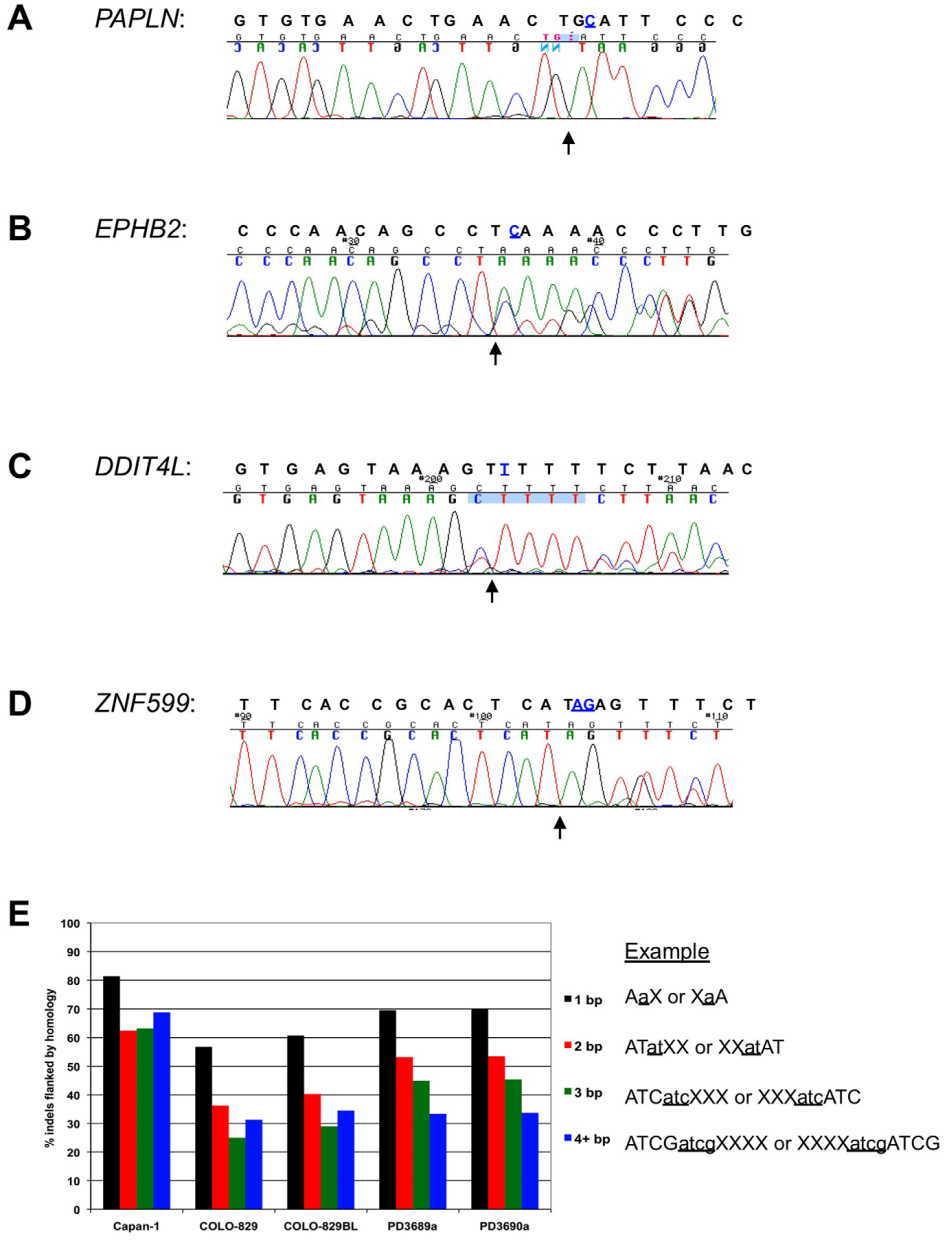
Indels identified in Capan-1. **A.** Chromatogram depicting the deletion of C (highlighted in blue) in *PAPLN* detected for Capan-1. The genomic reference sequence is shown above the chromatogram. The arrow indicates the position of the deletion. **B.** As A, for deletion of C in *EPHB2*. **C.** As A, T>C SNV and deletion of T in *DDIT4L*. **D.** As A, deletion of AG in *ZNF599*. **E.** Comparison of the percentage of indels of 1, 2, 3, or >4 bp length that are flanked by homology in Capan-1, COLO-829, COLO-829BL, PD3689a, and PD3690a. Homology is defined as having the same sequence as the inserted or deleted bases either immediately 5′ or 3′ to the indel, as represented by the examples.

A subset of the high-confidence coding frameshifts was validated by PCR and conventional Sanger sequencing, using a biological replicate genomic DNA sample. Eight novel frameshifts (homozygous: *PAPLN*; Heterozygous: *EPHB2*, *LRRC7*, *DDIT4L*, *ADD3*, *SF1*, *C17orf57*, and *ZNF599*) present in Capan-1 but not detected in the HapMap genomes were confirmed by Sanger sequencing, in addition to a further 17 frameshifts and 10 three base-pair indels common to both Capan-1 and HapMap. All of the novel frameshifts, as well as the in-frame indels, were authenticated by Sanger sequencing (Fig. 3A–D and data not shown). Five of the low-rate frameshifts that failed to pass the stringent filters could not be confirmed, supporting the validity of our analysis. Some of the genes affected by frameshifts, such as *EPHB2*, have been previously associated with tumourigenesis in other organs [58], raising the possibility that a number of these may represent novel driver mutations.

### Sequence context of indels

It has been hypothesized that defects in homologous recombination, such as those resulting from loss of BRCA2 function, may lead to an increase in small deletions [10], [59]. These may be flanked by repetitive sequences, if alternative processes of DNA double-strand break repair, such as non-homologous end joining or single-strand annealing are involved in the restoration [10], [15], [59]. On this basis we examined the immediate sequence context of the small indels detected in the Capan-1 genome, and compared these to the two *BRCA2*-deficient tumours sequenced in the Stephens dataset [27], in addition to the *BRCA*-proficient COLO-829 and COLO-829BL matched pair [36]. Indels were categorized by length (1, 2, 3, or 4+ bp), and scored for whether the flanking sequence either 5′ or 3′ to the indel was identical to the sequence inserted/deleted (Fig. 3E). In the Capan-1/HapMap comparison, more than 60% of the indels detected in Capan-1 were identical to the flanking sequence. This is significantly more than would be expected by chance alone (50, 12.5, 3, and 0.8% respectively for a 1, 2, 3, and 4 bp indel) and was considerably greater than observed in the in COLO-829/COLO-829Bl comparison, where presumably HR is active. Capan-1 exhibits a greater frequency of indels associated with homology than COLO829 despite the much higher depth of genomic sequence generated for COLO829. Hence the observations made for Capan-1 are unlikely to be an artefact resulting from the differences in sequencing depth. Further, the association between indels and flanking homology remains high for all lengths of indels in Capan-1, which contrasts with the other cell lines (Fig. 3E). The *BRCA2*-deficient tumour samples PD3689a and PD3690a have lower frequencies of indels flanked by homology, although it is possible that these rates are under-represented due to the low sequence depth used in their analysis (Fig. 3E). Taken together, this observation suggests that *BRCA2*-deficient tumour cells may be predisposed to a more frequent incidence of indels in short repetitive sequences.

## Discussion

Here we have generated the first comprehensive genome sequence analysis of a *BRCA2*-deficient cell line. It also represents the first such sequence of a widely used cell line derived from a pancreatic tumour. This dataset will therefore prove useful in deciphering the genetic contribution to both *BRCA2*-deficient and pancreatic cancers.

Many of the most frequently studied cell lines, such as Capan-1, were derived from patient tumour samples decades ago, and as such lack matched normal controls that can be used to determine germline variation. This study demonstrates that massively parallel sequencing can be utilized effectively even in the study of such cell lines. Our novel approach to filtration of the large variant dataset, using a combination of the publicly available databases in combination with HapMap samples prepared, sequenced, and analysed in an analogous manner to the query, will provide a strategy for the future evaluation of many more cell lines. Detailed genomic characterization of these lines will be beneficial, not only as a comparative resource for the large-scale tumour sequencing studies, but also to assist in deciphering many unresolved molecular and cellular observations.

Most rearrangements detected in Capan-1 are intrachromosomal, as in many breast tumours [27]. As would be expected, the rearrangements are spread throughout the genome and therefore reflect the patterns seen in other solid tumours [24], [27], [60], rather than as hotspots of rearrangement observed recently in leukaemia [61]. Interestingly, Capan-1 exhibits a greater overall number of rearrangements than has been previously described in other tumour types, including breast, which often show high levels of chromosome rearrangement [24], [27], [36], [60]. It seems possible that this is reflective of the genomic instability that results from the loss of BRCA2 function in HR. However, our analysis shows that, to date, there are too few complete sequences of HR-deficient genomes to be able to prove this with statistical significance (Fig. 1).

Capan-1 is a particularly intriguing cell line as it is both *BRCA2* deficient, like many familial breast cancers [58], yet derived from a pancreatic adenocarcinoma. Although two *BRCA2*-deficient tumours have previously been assessed by massively parallel DNA sequencing, this was only at low depth, sufficient for studies of chromosomal rearrangements, but not SNPs and indels [27]. Previous genome-wide SNP analyses of pancreatic cancers using microarrays suggested that a small number of signalling pathways and cellular processes are altered in most pancreatic tumours [62]. Many of these core processes are also affected by the novel variations detected here in Capan-1, including apoptosis (*DCC*), the DNA damage response (*ATM*), GTPase-dependent signalling (*SMAP2*), and Wnt signalling (*FZD10*), in addition to those already established, namely KRAS and TGFb signalling (the *KRAS* and *SMAD4* genes respectively). Interestingly, the recent study of genomic variation in metastases from pancreatic cancers [63] demonstrated that most homozygous mutations in the metastasis were already present in [58] parental tumour, hence were most likely to represent tumour suppressors. This is an important finding with respect to Capan-1 and other cell lines that were derived from a metastasis rather than the primary tumour.

The most common origin of SNPs in primates is through deamination of methyl-cytosine causing transition of cytosine to thymine [64]. Here we also observed that such C>T transitions constitute the most common type of base substitution in the Capan-1 genome. Base substitution frequencies have previously been analyzed in 24 advanced pancreatic adenocarcinomas [62] and 11 breast tumours [65], using large-scale PCR-based resequencing studies of protein-coding exons. Whilst C>T transitions also predominated in both tumour types, the pattern of substitutions differed between pancreas and breast. In pancreatic adenocarcinomas, the vast majority of substitutions were either C>T (53.8%) or C>A (16.6%), with all other classes each accounting for only 5–10% of the total [62]. In contrast, the spectra of breast tumour mutations comprised C>T (36.5%), C>G (28.1%), and C>A (15.1%), with far fewer substitutions at A or T bases [65]. We observed Capan-1 to be more akin to pancreatic adenocarcinomas in terms of the pattern of exome base substitutions, although A>G transitions were the second most common class of mutation (Fig. 2).

The observation that the incidence of small indels in the context of short regions of repetitive sequence occurs more frequently in Capan-1, and to some extent in the BRCA2 deficient tumours PD3689a and b (Fig. 3), is intriguing. Such a signature may well indicate the use of alternative pathways of DNA double strand break repair, such as non-homologous end joining or single-strand annealing [10], [15], [59], to compensate for the lack of HR. With the future sequencing of further BRCA deficient genomes, it will be possible to decipher whether this is in fact a *bone fide* DNA signature representative of a cellular defect in HR, which might be used as a biomarker to identify patient populations that might benefit from targeted therapies such as PARP inhibitors [66].

This comprehensive sequence analysis of a *BRCA2*-deficient pancreatic cancer cell line provides a valuable resource that will, in combination with large-scale genome resequencing of patient tumour samples, facilitate the identification of new biomarkers and targets for therapy. The compilation of such genomic datasets will undoubtedly underlie a greater understanding of this complex disease, and how loss of *BRCA2* contributes to tumour progression.

## Methods

### Genomic DNA isolation

Genomic DNA was isolated from asynchronous Capan-1 cells (ATCC Manassas, Virginia, USA) using the Gentra PureGene kit (Qiagen), according to the manufacturers instructions. DNA was quantified using the Quant-It PicoGreen kit (Invitrogen).

### Array CGH

aCGH was performed using a 32K BAC re-array collection (CHORI) tiling path platform, constructed at the Breakthrough Breast Cancer Research Centre, as previously described [67], [68].

### Whole genome sample preparation

Four micrograms genomic DNA was fragmented to 500 bp using a Covaris E Series instrument (Covaris Inc.). PCR-free libraries were then prepared using the Illumina Paired-end DNA sample prep kit, according to [22]. Final libraries were quantified using a Bioanalyzer DNA chip (Agilent) and subsequent qPCR.

### Exome sample preparation

Four micrograms genomic DNA was fragmented to 200 bp using a Covaris E Series instrument (Covaris Inc.). Paired-end libraries were prepared using the Next DNA sample preparation reagent set (New England Biolabs) with custom primers. The library was then hybridised to the 38 Mb SureSelect Human All Exon kit (Agilent), according to the manufacturer's instructions. Final libraries were quantified using a Bioanalyzer DNA chip (Agilent) and subsequent qPCR.

### Massively Parallel Sequencing

Sequencing reactions were performed on an Illumina GAIIx genome analyser using a paired-end 2x 76 bp strategy, according to the manufacturers guidelines.

### Bioinformatic analysis

#### Alignment

Prior to alignment, an in-house perl script was used to filter the raw reads in order to remove identical mate pairs that could represent PCR duplicates, as well as those in which one or both of the reads contained a highly repeated motif or a high number of uncalled bases (>5N). The alignment against human genome version 19 (ensembl 56) was performed using BWA [23]. Alignment of reads with greater than 4 mismatches compared to the reference genome was not permitted, reporting only those reads with up to 5 equal-alignment-quality hits. To facilitate detection of small indels and SNPs, a combination of both mismatches and up to one gap in a single read was permitted, using default penalty parameters.

SAMTools [30] was utilized for both post-alignment filtering and the calling of SNVs. The *rmdup* option was used to assure that PCR duplicates had been completely discarded, and soft-clipped reads were also removed. The *pileup* option was used to call SNVs and obtain details of coverage and depth. Further analysis was performed using in-house scripts (detailed below). Larger indels were detected using Pindel [31].

#### Detection of SNVs and small indels

Analysis was performed on the exome rather than whole genome data as we were most interested in the identification of coding mutations, and as the exome had been sequenced to a much higher depth thus precluded structural false positives. The exomes of four genotyped normal genome HapMap samples (NA11881 (male); NA12761, NA12813, and NA12892 (female)) were sequenced and analysed in an identical manner to Capan-1 in order to normalize for copy number variation and filter for common genome polymorphisms. All variants detected in the HapMap samples were disregarded in Capan-1 as these were most likely to be false positive (for technical or misalignment reasons) or non-somatic. The remaining variants were subsequently filtered. The aCGH data was used to estimate copy number status of each genomic region, and this was incorporated into the filtration. Heterozygous variants in single copy regions were discarded, and elsewhere, a minimum number of reads bearing the variant allele per copy was required. The identification of indels based on alignment analyses is more biased that SNP identification, leading to different variant features. Hence, we used different filtering premises depending on whether the variant was a SNP or an indel, but took into consideration the copy number status in both cases.

For SNP filtering, the concordant genotypes for all four HapMap samples were used to establish that SNPs with a variant rate (number of reads bearing the variant allele as a fraction of the total number of reads in that position) greater then 0.88 or less than 0.10 (data not shown) should be considered as homozygous variants for variant and reference allele, respectively. We observed that the heterozygous variant rate fluctuated from 0.33 to 0.67 (data not shown). In order to discard false variants located in low depth regions (problematic regions), we applied a confidence threshold of 10 reads per genomic copy.

For indel filtering, variants with a variant rate greater than 0.81 were considered to be homozygous. A threshold of 10 reads per genomic copy was applied, and only those variants where the number of reads bearing the variant allele was 0.75x the number of reads estimated to correspond to one genomic copy were considered (calculated as the depth at each position divided by the copy number for that region, e.g. a minimum of 15 variant reads in a 3 copy region with a depth of 60).

After filtering processes, remaining variants were classified according to their functional consequences. We used an in-house perl script to extract this information from Ensembl (www.ensembl.org) using the PerAPI application, checking functional consequences of each variant in every affected transcript for the gene. We also distinguished between previously described and novel variants using this tool.

#### Structural variations

These were identified using BreakDancer [26] with default parameters. Filtering process was based on depth, keeping those rearrangements supported by at least 10 different mate pairs. For intrachromosomal rearrangements, insert size should be greater than 1 kb, according to the variability in the insert size produced by the technique. Capan-1 is a highly rearranged tumour cell line according to SKY karyotype [21], thus is not surprising that a high number of interchromosomal rearrangements passed the filtering process. As no normal match is available, we manually checked all regions involved in interchromosomal rearrangements in order to exclude those regions showing sufficient homology to represent a strong possibility of being false positives due to misalignments.

### Sanger sequencing

Sanger sequencing was used to validate our analysis pipeline using standard protocols (see also Table S9). Primers were confirmed to yield unique products from genomic DNA using the UCSC In Silico PCR tool [69].

## Supporting Information

Table S1 **Coverage variation in Capan-1 whole genome sequence.** Median, mean, minimum and maximum values of the depth are shown for each cytoband region. In addition, global median value of the depth for each chromosome and for the whole genome are shown.
(XLS)

Table S2 **Coverage variation in Capan-1 exome capture.** Median, mean and standard deviation values of the depth and coverage were calculated for the rbaited region for each chromosome and for the whole exome baited regions set.
(XLS)

Table S3 **Interchromosomal rearrangements correlating with genic regions.** Candidate interchromosomal rearrangements at genic sites are listed.
(XLS)

Table S4 **Intrachromosomal rearrangements correlating with genic regions.** Candidate intrachromosomal rearrangements at genic sites are listed.
(XLS)

Table S5 **Coverage variation in HapMap exome capture.** Sequence coverage from a WEC analysis in four HapMap samples is shown.
(XLS)

Table S6 **SNVs identified from exome resequencing of Capan-1.** Whole set of variants identified in CAPAN1 WEC data after the filtering process.
(XLS)

Table S7 **Novel SNVs identified in Capan-1 that correlate with genes listed in the Cancer Genome Census.** List of variants found in CAPAN1 located on cancer related genes, according to the Cancer Gene Census.
(XLS)

Table S8 **Indels indentified from exome resequencing of Capan-1.** List of indel variants identified in CAPAN1 WEC data after filtering process.
(XLS)

Table S9 **Number of SNVs and Indels validated by Sanger sequencing.** List of Sanger sequencing validated SNVs and Indels are shown.
(XLS)

Figure S1 **aCGH data for CAPAN1.** Copy number status was calculated based upon an aCGH analysis. Haploid regions were estimated to present a log2 value between −1 and −0.45, diploid regions a log2 value between −0.45 and 0.05, triploid regions a log2 value ∼0.05–0.30 and tetraploid regions a log2 value of ∼0.30–0.55. Regions presenting values greater than 0.55 were considered pentaploid for filtering purposes.
(PPT)
