# Supplementary material for: Comprehensive Genomic Analysis of a BRCA2 Deficient Human Pancreatic Cancer
Source: PLoS One. 2011 Jul 5;6(7):e21639. doi: 10.1371/journal.pone.0021639 (PMC3130048; doi:10.1371/journal.pone.0021639)
Supplement: Figure S1 — aCGH data for CAPAN1. Copy number status was calculated based upon an aCGH analysis. Haploid regions were estimated to present a log2 value between −1 and −0.45, diploid regions a log2 value between −0.45 and 0.05, triploid regions a log2 value ∼0.05–0.30 and tetraploid regions a log2 value of ∼0.30–0.55. Regions presenting values greater than 0.55 were considered pentaploid for filtering purposes. (PPT) [file pone.0021639.s010.ppt]

## Slide 1
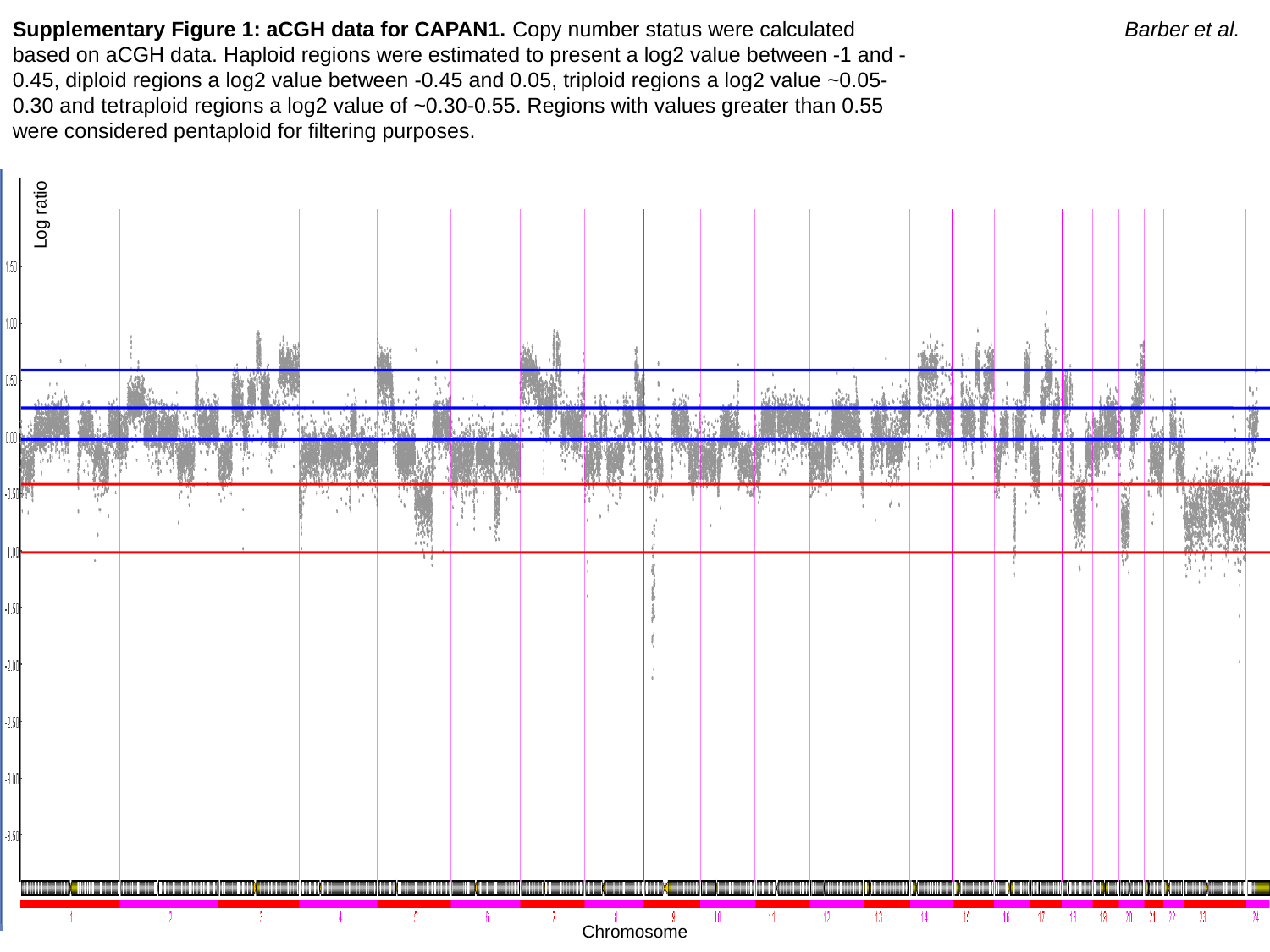

Barber et al.
Supplementary Figure 1: aCGH data for CAPAN1. Copy number status were calculated based on aCGH data. Haploid regions were estimated to present a log2 value between -1 and -0.45, diploid regions a log2 value between -0.45 and 0.05, triploid regions a log2 value ~0.05-0.30 and tetraploid regions a log2 value of ~0.30-0.55. Regions with values greater than 0.55 were considered pentaploid for filtering purposes.
Log ratio
Chromosome
